# CD109 released from human bone marrow mesenchymal stem cells attenuates TGF-β-induced epithelial to mesenchymal transition and stemness of squamous cell carcinoma

**DOI:** 10.18632/oncotarget.21067

**Published:** 2017-09-16

**Authors:** Shufeng Zhou, Renzo Cecere, Anie Philip

**Affiliations:** ^1^ Division of Plastic Surgery, Department of Surgery, McGill University, Montreal, QC, Canada; ^2^ Division of Cardiac Surgery, Department of Surgery, McGill University, Montreal, QC, Canada

**Keywords:** human bone marrow mesenchymal stem cells, squamous cell carcinoma, CD109, TGF-β, epithelial to mesenchymal transition

## Abstract

Although there is increasing evidence that human bone marrow mesenchymal stem cells (hBM-MSCs) play an important role in cancer progression, the underlying mechanisms are poorly understood. Transforming growth factor β (TGF-β) is an important pro-metastatic cytokine. We have previously shown that CD109, a glycosylphosphatidylinositol-anchored protein, is a TGF-β co-receptor and a strong inhibitor of TGF-β signalling. Moreover, CD109 can be released from the cell surface. In the current study, we examined whether hBM-MSCs regulate the malignant properties of squamous cell carcinoma cells, and whether CD109 plays a role in mediating the effect of hBM-MSCs on cancer cells. Here we show that hBM-MSC-conditioned medium decreases proliferation and induces apoptosis in human squamous carcinoma cell lines, A431 and FaDu. Importantly, hBM-MSC-conditioned medium markedly suppresses markers of epithelial-to-mesenchymal transition and stemness, and concomitantly decreases cell migration, invasion, and spheroid formation in A431 and FaDu cells. In addition, knockdown of CD109 in hBM-MSCs abrogates the anti-malignant activity of hBM-MSC-conditioned medium on A431 and FaDu cells. Furthermore, overexpression of CD109 in A431 cells decreases their malignant traits. Together, our findings suggest that hBM-MSCs inhibit the malignant traits of squamous cell carcinoma cells by a paracrine effect via released factors and that CD109 released from hBM-MSCs, at least partially, mediates these effects.

## INTRODUCTION

The incidence of non-melanoma skin cancer, including squamous cell carcinoma (SCC), has increased dramatically over the past decades, and is currently the most common neoplasia in Caucasian populations [1]. A subpopulation of cancer cells with stem cell-like properties known as cancer stem cells (CSCs) have been identified in several types of cancer including SCC, and are thought to be responsible for both therapeutic resistance and metastasis [2, 3]. Several studies have implicated epithelial-to-mesenchymal transition (EMT) in therapeutic resistance and the initiation of metastasis [4–7]. Transforming growth factor-beta (TGF-β), a potent inducer of EMT in the tumor microenvironment [8, 9], is upregulated in many human malignancies including SCCs [10]. However, TGF-β exerts paradoxical effects during carcinogenesis - acting as a tumor suppressor in the early stages of tumor progression and switching to a pro-metastatic signal during later stages [11–15]. In the late stages of tumor, cancer cells produce high levels of TGF-β or exhibit altered TGF-β signaling that can aberrantly activate the EMT process to promote tumor cell migration and invasion, generation of CSCs and initiation of metastasis [16, 17]. Because TGF-β is a central mediator of EMT and metastasis, there has been intense interest in targeting the TGF-β signalling pathway for anti-metastatic therapies [18].

Our group has identified CD109, a glycosylphosphatidylinositol (GPI)-anchored protein, as a TGF-β co-receptor and a potent inhibitor of the TGF-β signalling pathway [19–21]. Furthermore, our group has previously shown that CD109 is released from the cell surface and that both membrane anchored and released CD109 negatively regulate TGF-β signaling via different mechanisms [19–23]. CD109 expression has been shown to be dysregulated in many cancers [24–26], suggesting that CD109 may play a role in cancer progression. In SCC, an inverse correlation between CD109 expression and the grade of the cancer has been reported, with CD109-positive tumors being well differentiated and of a lower grade, whereas CD109-negative tumors being poorly differentiated and of a higher-grade [25, 27]. These reports suggest that CD109 may play a critical role in the regulation of cancer cell stemness and metastasis, and, importantly, that a reduction of CD109 levels may contribute to SCC progression.

Human bone marrow mesenchymal stem cells (hBM-MSCs) are non-hematopoietic stem cells found in bone marrow that are capable of self-renewal and multi-lineage differentiation. These multipotent cells not only can replicate as undifferentiated cells, but also can differentiate into mesenchymal tissues such as bone, cartilage, fat, tendon, muscle, and marrow stroma [28]. Although hBM-MSCs have initially gained attention due to their potential applications in tissue engineering and disease therapy, their homing potential to the tumor site and tropism in the tumor microenvironment led to a great deal of interest in their functional role in tumors [29–31]. By secreting a variety of cytokines having both paracrine and autocrine functions in the tumor milieu, hBM-MSCs exhibit multifaceted effects on tumor cells, which include the regulation of apoptosis and angiogenesis, as well as immunomodulation. However, there is much controversy in the literature regarding the role of MSCs in tumor progression in general, with several studies reporting tumor promoting effects while many others ascribe a tumor inhibitory role [30, 32–34]. This also holds true for BM-MSCs, as some reports demonstrate that BM-MSCs favor tumor growth *in vivo* [35, 36] while other studies suggest that BM-MSCs exert an anti-tumorigenic effect by inducing apoptosis or modulating the immune system [37, 38]. These discrepant results can at least in part be explained by the broad array of cytokines and other factors produced by BM-MSCs and the paucity of information regarding the complex interactions between BM-MSCs and tumor cells. Defining the molecular mechanisms underlying the interactions between MSCs and tumor cells within the tumor microenvironment may lead to novel therapeutic approaches in cancer treatment.

In the current study, we sought to determine whether hBM-MSCs regulate the malignant properties of SCC cells, and whether CD109 plays a role in mediating hBM-MSC's effects on tumor progression. Our findings indicate that hBM-MSCs inhibit the malignant traits of SSC cells by a paracrine effect via released factors and that the anti-cancer effect of hBM-MSC is at least in part due to CD109 released from hBM-MSCs. This is the first report suggesting that CD109 may account for the tumor inhibitory activity of hBM-MSCs and linking CD109 to the inhibition of TGF-β-induced EMT and stemness.

## RESULTS

### Conditioned media derived from human bone marrow mesenchymal stem cells (hBM-MSC-CM) decreases proliferation and induces apoptosis of SCC cells

**Figure 1:**
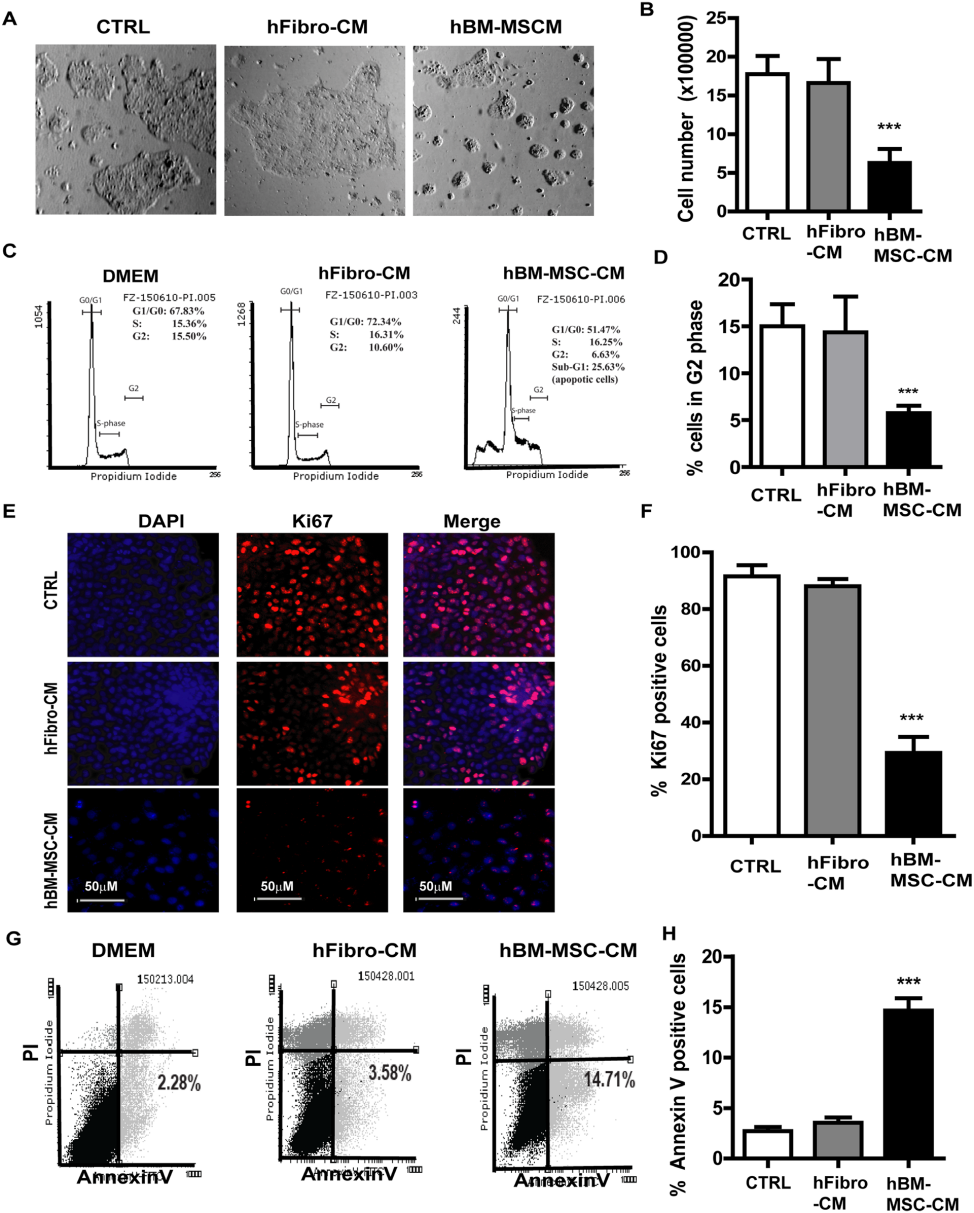
hBM-MSC-CM exhibits anti-proliferation and pro-apoptosis effect on SSCs **(A)** Phase contrast pictures and **(B)** cell count analysis of A431 cancer cells treated with hBM-MSC-CM, human fibroblast-CM (hFbrio-CM) and DMEM (CTRL) for 72 hrs. **(C-D)** Cell cycle analysis of A431 cells treated as described in (A) showed that hBM-MSC-CM significantly decreased A431 cells proliferation (cells in S and G2 phase). **(E-F)** Immunofluorescence microscopy of A431 cell treated as in (A) and stained for Ki67 (Red) and DAPI (blue) showed that hBM-MSC-CM significantly decreased Ki67 positive cells. **(G-H)** The A431 cancer cells were treated as in (A) and analyzed by Flow cytometry for apoptosis by Annexin V/PI staining on a FACSCalibur cytometer. hBM-MSC-CM significantly increased the number of apoptotic cancer cells (Annexin V positive and PI negative). All the results are shown as the mean ± SD of at least three independent experiments. Significance is calculated using a one-way ANOVA analysis. ^*^ *P* < 0.05, ^**^ *P* < 0.01 and ^***^ *P* < 0.001.

We first investigated the effect hBM-MSC-CM vs. conditioned medium from human fibroblast cells (hFibro-CM) on A431 cells. Cancer cells were cultured in hBM-MSC-CM, hFibro-CM and DMEM, respectively for 72 hours, then submitted to a cell number count and a cell cycle analysis. As expected, cell counting revealed that hBM-MSC-CM reduced the proliferation of A431 cells by about 3-fold. (Figure 1A and 1B). We also observed an arrest of the cell cycle associated with a reduction of cell proliferation (Figure 1C and 1D). hBM-MSC-CM generated a reduction of cell number in G2 (8.38% ±3.27 %,) and S phase (12.57% ±2.05%), respectively, while more cells entered Sub G1(apoptotic cells, 15.69%). Conversely, more cancer cells entered G2 (18.63% ±6.49%) and S phase (18.4% ±6.19%) when cultured in hFibro-CM and DMEM (Figure 1C and 1D). hBM-MSC-CM also induced a 60% decrease in the Ki67 proliferation marker expression in A431 cancer cells (Figure 1E and 1F). This suggests that hFibro-CM and DMEM (as controls) exhibit no inhibitory effects on cancer cell growth while hBM-MSC-CM exhibits inhibitory effect on skin cancer cells. Similar results were obtained from FaDu, a model cell line of a hypopharyngeal squamous cell carcinoma (Supplementary Figure 1A and 1B).

In order to investigate whether the inhibition of A431 cell growth is due to a growth delay or an increase of apoptosis (or both), we analyzed the apoptosis by flow cytometry and Annexin V/7-AAD staining (Figure 1G and 1H). A431 cells cultured in hBM-MSC-CM exhibited more apoptosis (14.71% ± 5.23% Annexin V+) relative to cells cultured in hFibro-CM (3.58 ± 2.45%) and DMEM control medium (2.28 ± 4.45%,). This is consistent with the observation of an increased sub-G1 peak (25.69% ± 5.51%) in cells cultured in hBM-MSC-CM (Figure 1C and 1D). Conversely, the sub-G1 peak was markedly reduced in A431 cells treated with hFibro-CM (2.30 ± 3.45%) and DMEM (2.49± 4.05%) (data not shown). These results suggest that the anti-proliferative and pro-apoptotic effect is therefore specific to the hBM-MSC-CM as hFibro-CM and DMEM showed no such effect on A431 cells. Similar results were obtained from FaDu, a model cell line of a hypopharyngeal squamous cell carcinoma (Supplementary Figure 1C and 1D).

### hBM-MSC-CM reduces epithelial-mesenchymal transition (EMT) markers in A431 cells

**Figure 2:**
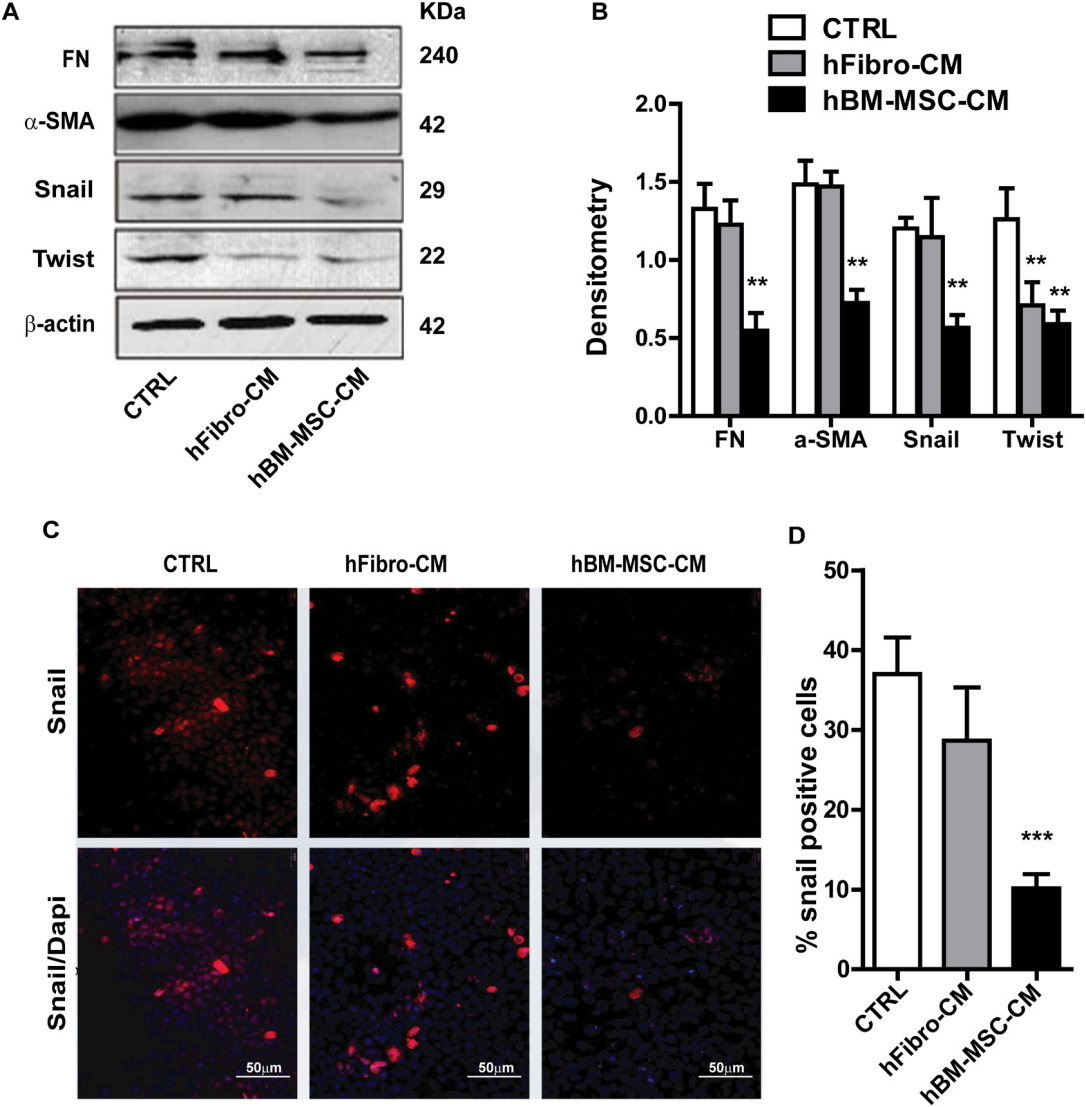
hBM-MSC-CM decreases EMT traits of SCCs **(A** and **B)** Western blot analysis of A431 cancer cells treated with hBM-MSC-CM, human fibroblast-CM (hFibro-CM) and DMEM (CTRL) for 72 hrs. Representative blot images using the indicated antibodies (A) and the quantification (B). hBM-MSC-CM significantly decreased the EMT markers. **(C** and **D)** Immunofluorescence microscopy of A431 cell treated as in (A) and stained for Snail (Red) and DAPI (blue) showed that Snail positive cells are decreased upon treatment with hBM-MSC-CM compared to controls. All the results are expressed as the mean ± S.D. of three independent experiments. Significance is calculated using a One-Way ANOVA ^*^ P < 0.05. ^**^ P < 0.01 and ^***^ P < 0.001. Magnification, ×100.

Epithelial-mesenchymal transition (EMT), a driving force of metastasis [5, 39], is employed by cancer cells to enhance migration and invasion, and to generate cancer stem cells [40–42]. To evaluate whether, and if so, how hBM-MSC-CM modulate the EMT response, we measured the expression of EMT markers in A431 cancer cells untreated or treated with hBM-MSC-CM. Compared to hFibro-CM and DMEM, hBM-MSC-CM significantly attenuated the expression of the EMT transcription factors Twist and Snail (Figure 2A and 2B). To further confirm this finding, cells were grown as monolayer cultures and immunostained to measure the expression of the EMT master regulator Snail. hBM-MSC-CM treatment markedly reduced the percentage of Snail positive cells (Figure 2C and 2D), which is consistent with the observed ability of hBM-MSC-CM to suppress EMT. Through western blot analysis, we also demonstrated that hBM-MSC-CM decreased the expression of the mesenchymal proteins fibronectin (FN) and α-SMA (Figure 2A and 2B). Taken together, these results indicate that hBM-MSC-CM inhibits several master regulators of EMT and mesenchymal traits, such as fibronectin and α-SMA.

### hBM-MSC-CM suppresses migration and invasiveness of SCC cells

**Figure 3:**
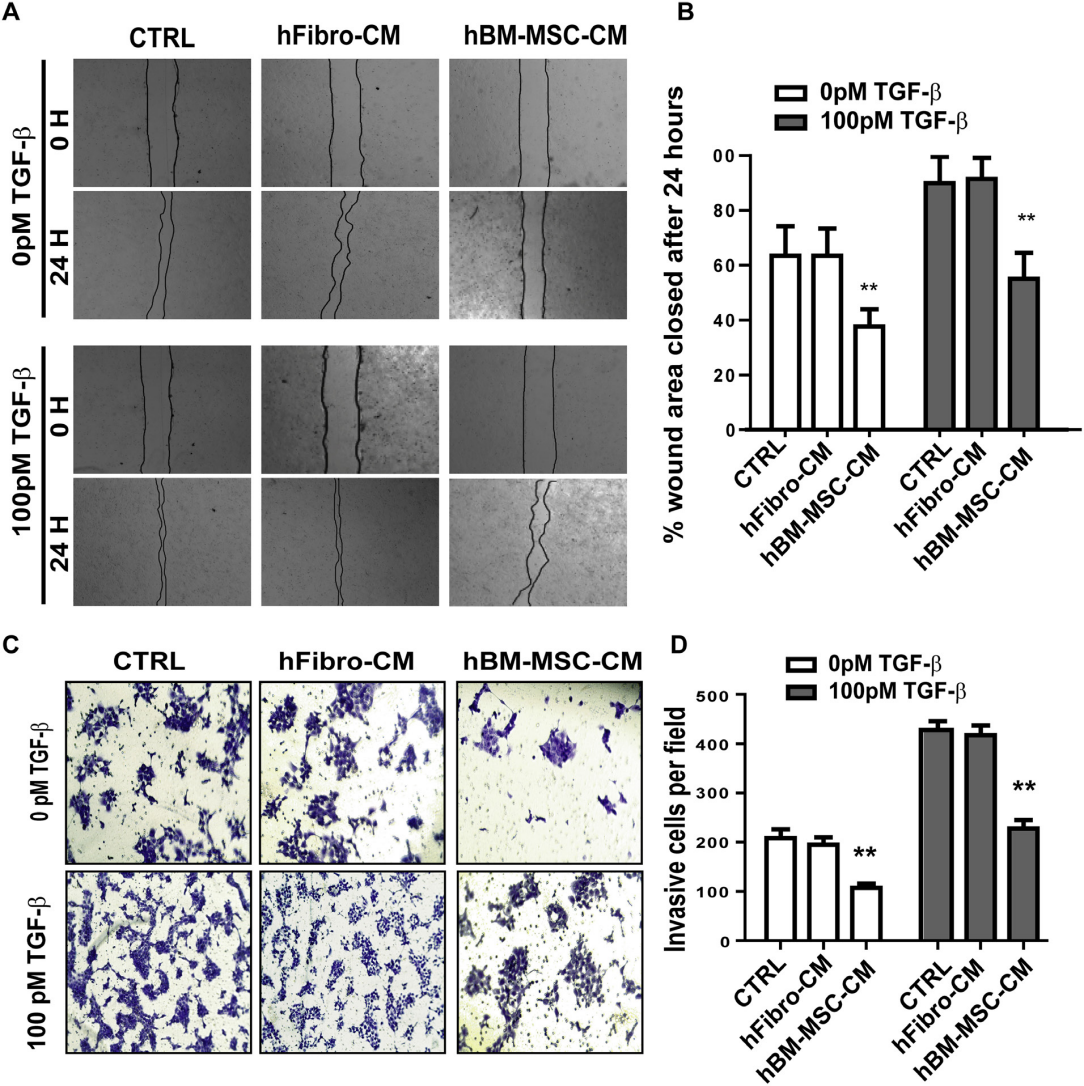
hBM-MSC-CM inhibits the migration and invasiveness of SCCs **(A-B)** Representative images (A) and quantification (B) of wound-healing assays on A431 cells treated as in Figure 1. Cell migration is expressed as a percentage of the scratch area filled by migrating cells at 24 h post scratch: migration rate = (T0 hr scratch width - T24 hr scratch width)/T0 hr scratch width) ×100%. hBM-MSC-CM significantly supressed cancer cell migration. **(C-D)** Representative images (C) and quantification (D) of an invasive assay done on equal number of A431 cancer cells treated as in (A). 10,000 cells were seeded on a BioCoat™ Matrigel^®^ Invasion Chamber for 24 hours. Cells that invaded through the matrigel-coated membrane were stained with 1% crystal violet, photographed, and counted. hBM-MSC-CM significantly supressed cancer cell invasion. All the results are expressed as the mean ± S.D. of three independent experiments. Significance is calculated using a one-way ANOVA; ^*^ *P* < 0.05. ^**^ *P* < 0.01 and ^***^ *P* < 0.001. Magnification, ×100.

Since enhanced cellular migration is a hallmark of metastatic cancer cells, we then performed *in vitro* wound healing assays to evaluate the effect of hBM-MSC-CM on the migration of SCC cells. In both the presence and absence of TGF-β, hBM-MSC-CM decreased the wounded area 24 hours post wounding in A431 cells as compared to those of hFibro-CM and DMEM (Figure 3A and 3B). At time zero, the scratch wounds were almost the same size in each experimental group, but the healing and cell migration rates were significantly reduced after 24 hours in hBM-MSC-CM treated cells compared to control medium and hFibro-CM (Figure 3A and 3B). As expected, in the presence of 100 pM of TGF-β, the cell healing rate was markedly faster than in the absence of TGF-β (Figure 3A and 3B). However, cells treated with the hBM-MSC-CM migrated to the wound area at a significantly slower rate than cells treated with control media. In the control groups, wounds were almost closed 24 h after scratching, whereas in the hBM-MSC-CM-treated group, the wound still remained considerably open, although significantly smaller than cells treated without TGF-β stimulation (Figure 3A and 3B). Similar results were obtained for FaDu cells(Supplemental Figure 2A and 2B). Together, these results indicate that hBM-MSC-CM can suppress the migration of SCC cancer cells *in vitro* by antagonizing TGF-β-induced cell migration.

Invasiveness of tumor cells is a defining step in the tumor progression and the ability to invade is one of the hallmarks of metastasis. To determine the effect of hBM-MSC-CM on the invasiveness of SCC cells, we carried out matrigel invasion assays. Cells treated with hBM-MSC-CM exhibited a 2.5-fold reduction in invasiveness compared to cells treated with either hFibro-CM or DMEM, regardless of the presence or absence of added TGF- β (Figure 3C and 3D). As expected, treatment with TGF-β significantly promoted invasiveness. Importantly, hBM-MSC-CM suppressed TFG-β–mediated cancer cell invasion, leading to a 2-fold reduction in invasion (Figure 3C and 3D). Similar results were obtained with FaDu cells (Supplementary Figure 1E and 1F). Overall, these results indicate that hBM-MSC-CM can decrease the invasiveness of SCC cells, which is consistent with the decrease in EMT response by hBM-MSC-CM, as well as the cell migration assay results.

### hBM-MSC-CM treatment decreases expression of the stemness marker SOX2 and concomitantly reduces tumor spheroid formation of SCC cells

**Figure 4:**
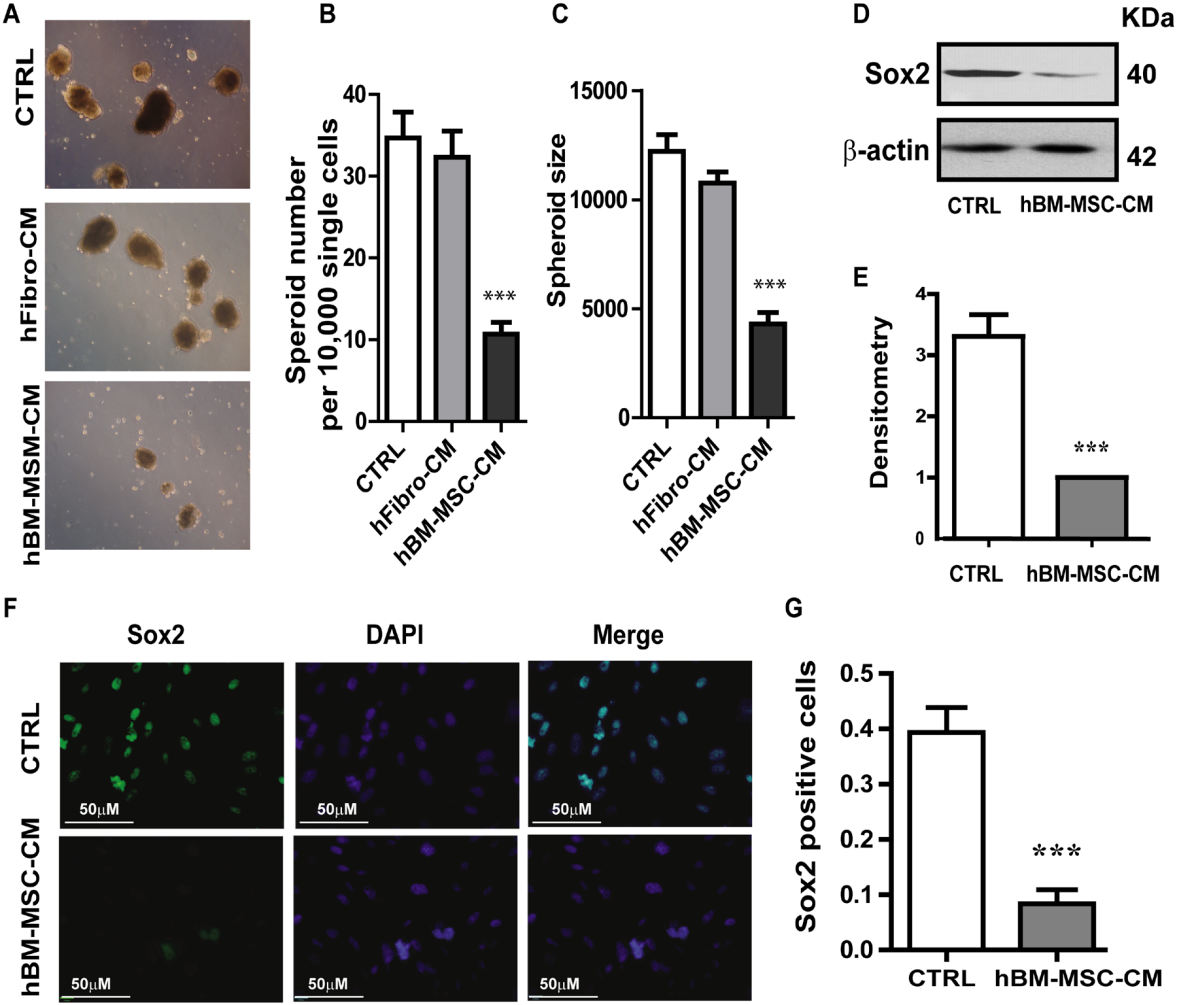
hBM-MSC-CM decreases SOX2 expression and tumor spheres formation **(A, B** and **C)** Tumor Spheroids Assay: (A) Representative images of tumor spheroids Quantification of (B) the number of tumor spheroids and (C) the size of tumor spheroids. Five random fields (x100) were photographed, and the number and the size of tumor spheroids were analysed. hBM-MSC-CM significantly supress tumor spheroid formation. A431 cell were treated for 72hrs with hBM-MSC-CM, hfibro-CM and DMEM, respectively, then 10,000 single cells will be plated on the ultra-low attached 6-well plate for the spheroid assay. **(D-E)** Representative image of Western blot analysis of SOX2 in A431 cell lines treated with hBM-MSC-CM or DMEM (CTRL). (E) Densitometry analysis of SOX2 related to Actin. hBM-MSC-CM dramatically decreased SOX2 expression. **(F-G)** Representative images of immunofluorescence staining of SOX2 in A431 cancer cells treated as in (D). (G) Quantification of SOX2 positive cells in the experiment of (F). hBM-MSC-CM significantly decreased the SOX2 positive cells. All the data are shown as means ± SD for at least three independent experiments. Significance is calculated using student-t test or a one-way ANOVA. ^*^P<0.05, ^**^P<0.01 and ^***^ P<0.001

Since tumorigenicity reflects the number of cancer stem cells, we evaluated the effect of hBM-MSC-CM on *in vitro* tumorigenicity and the self-renewal potential of A431 cancer cells. Because the ability to form tumor spheres *in vitro* depends on the presence of self-renewing stem cells within the cancer cell population [43], we performed a Spheroid formation assay to serve as an *in vitro* surrogate measurement of tumorigenicity. We found that cancer cells treated with hBM-MSC-CM produced not only significantly lower number of spheres but also spheres of small size, compared to cells treated with the control media (Figure 4A, 4B and 4C). Measurements of the tumor sphere size demonstrate that cells treated in either DMEM or hFibro-CM formed robust tumor spheres with a mean diameter ranging between 300- 360 μm (Figure 4C). In contrast, cells treated with hBM-MSC-CM formed not only fewer spheres (2.5-fold decrease) (Figure 4A and 4B), but also significantly smaller sized spheres (<120 μm) (Figure 4C). Similar results were obtained with FaDu cells (Supplementary Figure 1G and 1H). Our results indicate that hBM-MSC-CM decreases cancer stem cell population.

Given that SOX2 is the most up-regulated transcription factor in squamous skin tumors and regulates self-renewal of stem cells [44, 45], we examined the expression of SOX2 in A431 cells untreated or treated with hBM-MSC-CM. Treatment with hBM-MSC-CM resulted in a reduction in SOX2 protein level, as detected by Western blotting (Figure 4D and 4E). This was further confirmed by immunofluorescence analysis, which showed that cells treated with hBM-MSC-CM exhibited a four-fold reduction in SOX2-positive cells (Figure 4F and 4G). These results demonstrate that treatment of SCC cells with hBM-MSC-CM significantly decreases SOX2 expression compared to treatment with either hFibro-CM or control media. Our results strongly suggest that hBM-MSC-CM exhibits a stemness-inhibiting function and can hinder the tumorigenicity of SCC cells *in vitro*.

### CD109 partially accounts for the anti-cancer effect of human BM-MSC-CM on SCC cells

**Figure 5:**
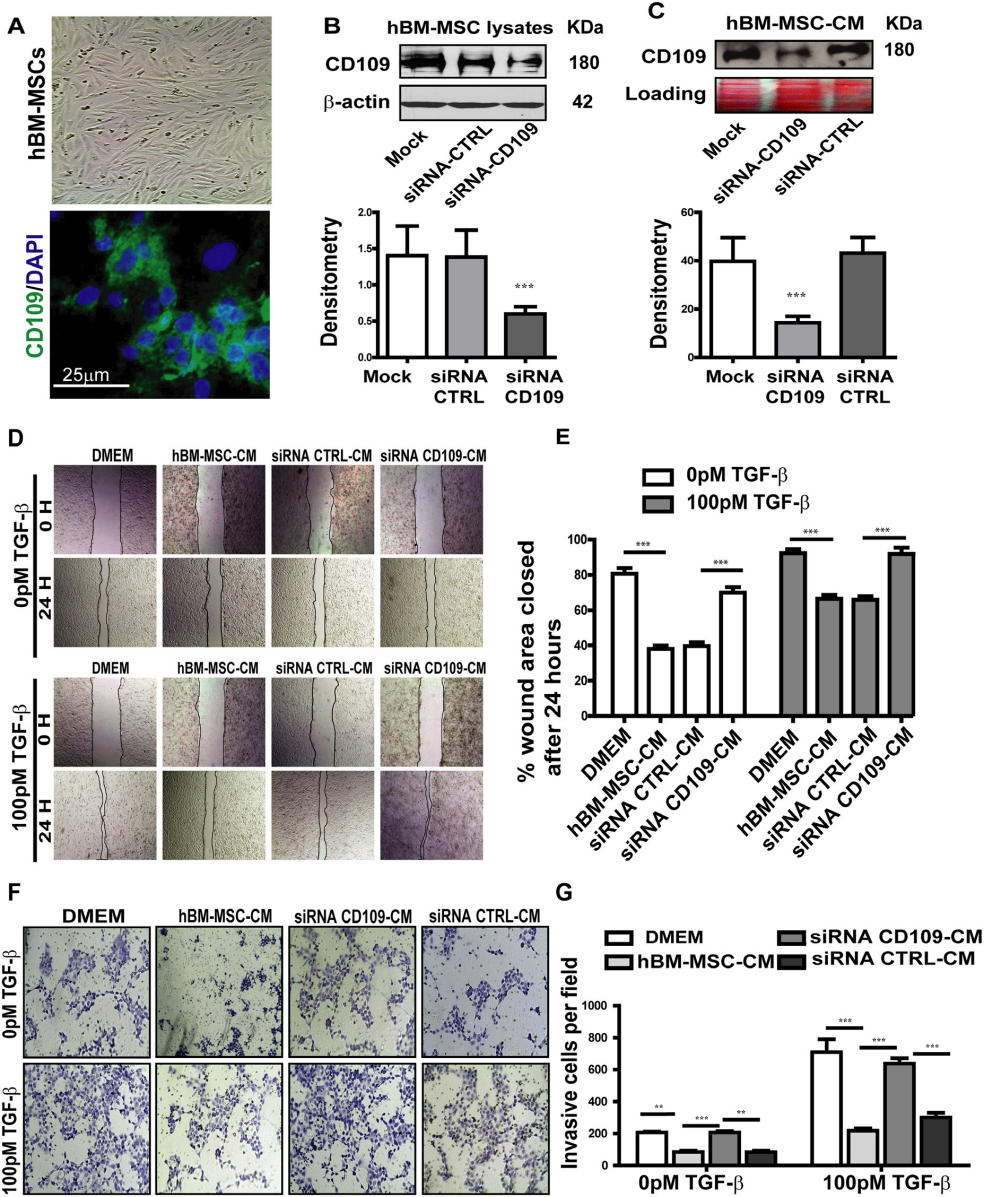
Knockdown of CD109 by targeted siRNA in hBM-MSCs significantly abrogated the anti-migration and anti-invasion of hBM-MSC-CM **(A)** Representative images of human BM-MSCs, Top: phase contrast image; Bottom: immunofluorescence image showing the expression of CD109. **(B-C)** Western blot analysis of CD109 expression in hBM-MSCs cell lysate (B) or conditioned medium (C) treated with CD109-siRNA or scrambled siRNA (siRNA-CTRL). CD109-siRNA significantly reduces CD109 expression in both cell lysates and conditioned medium confirming the efficiency of the knockdown. **(D-E)** Wound healing assay of A431 cancer cells treated with the indicated conditioned media. siRNA-mediated knockdown of CD109 in hBM-MSCs abrogates the anti-migration effect of hBM-MSCs-conditioned media. (D) Representative images (x100 magnification) and (E) quantification of the wound healing assay. **(F-G)** Matrigel Invasion Assay of A431 cancer cells treated as in (D). siRNA-mediated knockdown of CD109 in hBM-MSCs reduces the anti-invasiveness effect of hBM-MSCs-conditioned media. (F) Representative images (x100 magnification) and (G) quantification of the Matrigel invasion assay. Invading cells were stained with 1% crystal violet and counted under a microscope. All data are presented as the mean ± SD of at least three independent experiments. Significance is calculated using a One-Way ANOVA. ^**^ *P*<0.01 and ^***^ *P*<0.001.

Human BM-MSCs express CD109 as evaluated by confocal microscopy and western blot analysis (Figure 5A, bottom and 5B). CD109 was also detected in hBM-MSC-CM (Figure 5C), suggesting an active release of this protein by these cells. To investigate whether the anti-tumor activity of hBM-MSC-CM could be dependent on the released CD109 in the conditioned medium, we knocked down CD109 in hBM-MSCs using small interfering RNA (siRNA). Western blot of cell lysates confirmed that 80% reduction of CD109 expression was achieved in hBM-MSCs (Figure 5B). Of note, less than 30% of CD109 was detected in CM derived from CD109 knockdown hBM-MSCs, demonstrating a significant reduction of CD109 in the conditional medium from CD109-knockdown hBM-MSCs (Figure 5C).

We subsequently investigated the biological activity of CM derived from the CD109-knockdown hBM-MSCs. In the wound healing assay, as expected, cells treated with hBM-MSC-CM or with CM derived from hBM-MSCs transfected with the scramble siRNA exhibited a significantly slower closure of the wound area (Figure 5D and 5E), indicating a notable reduction in cellular migration. However, knocking down CD109 with its specific SiRNA significantly reduced the anti-migration activity of hBM-MSC-CM, as demonstrated by a significantly faster closure rate of the wound area, as compared to cells treated with CM derived from hBM-MSCs transfected with scramble siRNA (Figure 5D and 5E). The same phenomenon was observed in both the presence and absence of TGF-β stimulation (Figure 5D and 5E). The impact of CM derived from CD109-knockdown hBM-MSCs on invasion was then measured by matrigel invasion assays. The capacity of A431 cells to invade through matrigel was measured after treatment with CM from untransfected hBM-MSCs or CM from hBM-MSCs transfected with CD109 specific siRNA or control siRNA. Invading cell numbers were significantly higher in cells treated with CD109-KD hBM-MSC-CM when compared to cells treated with either hBM-MSC-CM or scrambled siRNA-hBM-MSC-CM. Crystal violent staining demonstrated that significantly higher number of cells passed through the matrigel coated membrane, as 57.3% invasive cells were detected when cancer cells were treated with CM derived from hBM-MSCs transfected with CD109-specific siRNA, while only 27.3% and 28.7% invasive cells were detected in cells treated from un-transfected hBM-MSCs and from hBM-MSCs transfected with Control siRNA, respectively. P<0.05 (Figure 5F and 5G). Therefore, the invasion of cells through the matrigel was significantly rescued when cells were treated with CM derived from CD109-knockdown hBM-MSCs, and almost reached the same level as that in the DMEM control group (Figure 5F and 5G). This is consistent with the results presented in Figure 4 and suggests that blocking CD109 in hBM-MSCs abrogates the anti-migration and anti-invasion biological effect of the conditioned media. Altogether, these results strongly suggest that CD109 released into hBM-MSC-CM is at least partially responsible for the suppression of cancer cell migration and invasiveness, since the knockdown of CD109 in hBM-MSC abrogates the anti-tumor activities of hBM-MSC-CM.

### Overexpression of CD109 in A431 cells effectively attenuates TGF-β-induced EMT response, migration and invasion

**Figure 6:**
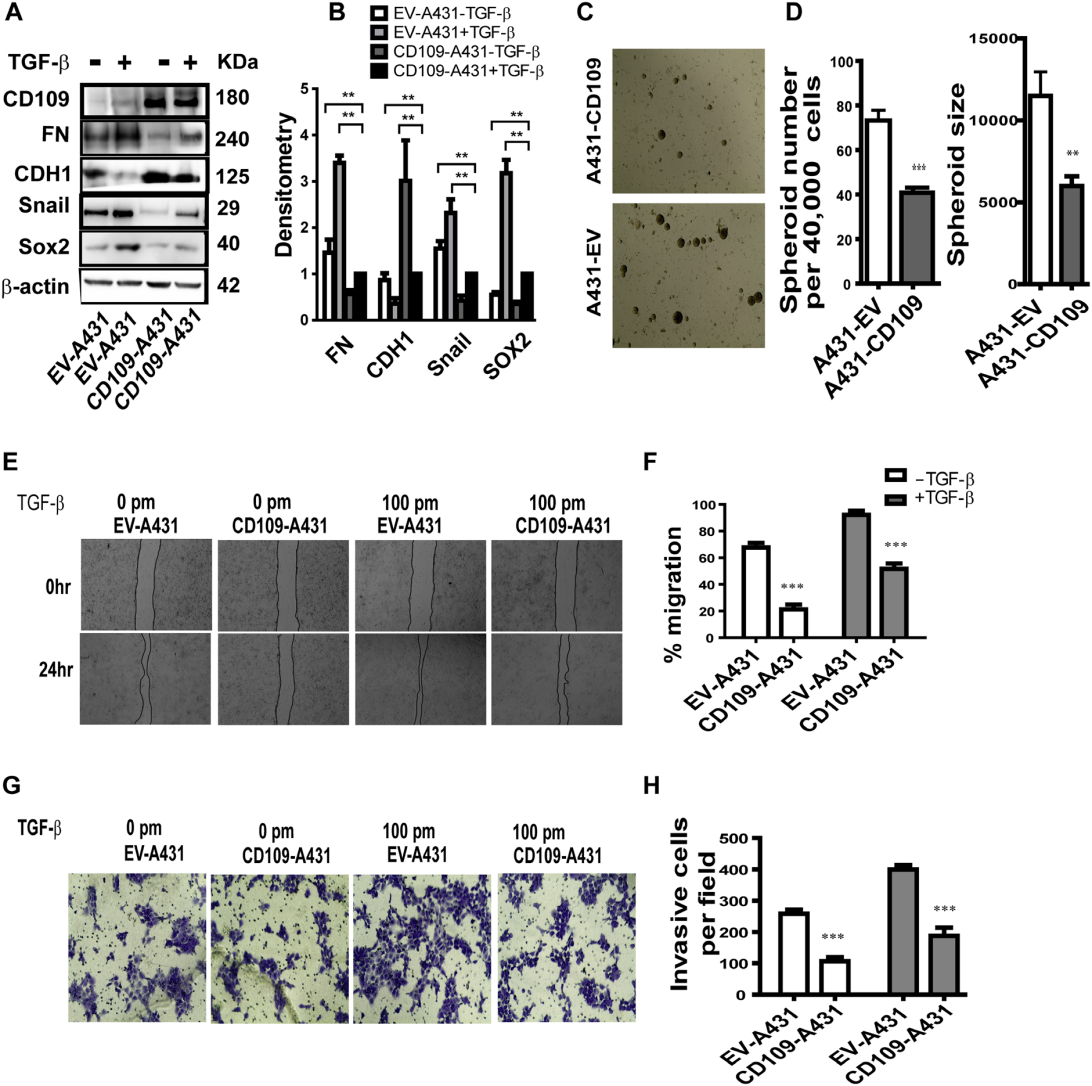
The effect of the overexpression of CD109 on A431 cells **(A-B)** Representative image of Western blot (A) for the indicated antibodies under the indicated conditions. (B) Quantification by densitometry of (A) with β-actin serving as a loading control. Overexpression of CD109 effectively attenuates cancer cells EMT response and Sox2 expression. **(C-D)** Tumor sphere formation assay. (C) Representative images of tumor spheroids; (D) Quantification of the number of tumor spheroids and the size of tumor spheroids. 40,000 cells were seeded and after 14 days, total spheres were counted. The size of spheroids was also analysed. CD109 overexpression significantly inhibits tumor sphere forming capacity of cancer cells. **(E-F)** Wound healing assay indicates that CD109 overexpression supresses A431 cancer cell migration. (E) Representative images of wound healing assay. (F) Quantification of the wound healing assay presented in (E). **(G-H)** Matrigel invasion assay indicates that CD109 overexpression inhibits A431 cancer cell invasion. (G) Representative images of matrigel invasion assay. (H) Quantification of the Matrigel invasion assay (100X) from (G). The invading cells were stained with 1% crystal violet. Invasive cells were counted under a microscope and 10 fields were counted for each experiment. All the data are presented as means ± SD of at least three independent experiments. Significance is calculated using a One-Way ANOVA. ^*^ P<0.05, ^**^ P < 0.01 and ^***^ P < 0.001.

To further confirm that CD109 is involved in the repression of EMT and stemness, we overexpressed CD109 in A431 cells [19]. We initially investigated the expression of several well-known EMT transcription factors and found that overexpression of CD109 resulted in the decreased expression of Fibronectin (FN, a mesenchymal protein), Snail (a master transcription regulator of EMT), and an increase of E-cadherin expression (CDH1, an epithelial marker) in the presence and absence of TGF-β (Figure 6A and 6B). As EMT activation can promote stemness, we next analysed whether CD109 overexpression regulates stemness. To this end, we examined whether CD109 overexpression affects Sox2 expression, known to be important for stem cell self-renewal. We found that CD109 overexpression resulted in decreased expression of SOX2 (Figure 6A and 6B), indicating that CD109 possesses a stemness-inhibiting capacity. Moreover, the capacity to form spheroids was markedly decreased in CD109 overexpressing cells compared to the empty vector transfected cells (Figure 6C and 6D). Altogether, our results indicate that overexpression of CD109 in SCC cells led not only to a reduction in EMT marker expression, but also to a decrease in Sox2 expression and spheroid formation, indicating a reduction in the cancer stem cell population.

Considering that TGF-β-induced EMT could increase cell motility and invasiveness, we then examined whether the overexpression of CD109 could modulate migration and invasion of A431 cells using a migration assay and a matrigel invasion assay, respectively (Figure 6E and 6F). As expected, cells treated with TGF-β migrated more efficiently, thereby closing the wound markedly faster than in the absence of TGF-β (Figure 6E and 6F). Importantly, CD109 overexpressing cells exhibited significantly slower wound closure compared to the cells transfected with the empty vector in both the presence and absence of TGF-β (Figure 6E and 6F). In addition, the matrigel gel invasion assay demonstrated that CD109-overexpressing cells exhibited a markedly reduced invasion compared with empty vector transfected controls (Figure 6G and 6H). Altogether, these results suggest that CD109 inhibits cancer cell migration and invasion.

## DISCUSSION

Mesenchymal stem cells including BM-MSCs have been extensively studied for their potential use as tools for tissue regeneration and gene delivery [46, 47]. The recent finding that BM-MSCs can also be recruited by tumor cells has generated an immense interest in understanding their potential role in cancer progression [31]. In the current study, we demonstrate that hBM-MSCs inhibit the malignant traits of SCC cells in a paracrine manner through the release of soluble factors. HBM-MSC-CM suppresses SCC cell proliferation, induces SCC apoptosis, and more importantly, hBM-MSC-CM inhibits EMT, migration, invasion, stemness and *in vitro* tumorigenicity in SCC cells. Moreover, siRNA-mediated knockdown of CD109, a TGF-β co-receptor and potent inhibitor of TGF-β signaling, reveals a critical role of CD109 in mediating the anti-cancer effects of hBM-MSC-CM.

Our results demonstrating that hBM-MSC-CM exhibits anti-proliferative and pro-apoptotic effects on A431 and FaDu SCC cells suggests that hBM-MSCs exert anti-cancer effects on SCC cells via releasing soluble factors. Our finding that hBM-MSC-CM markedly supresses the expression of several master regulators of EMT such as Twist, Snail and Slug and other mesenchymal traits in SCC cells (A431 and FaDu) suggests that the anti-cancer effects of hBM-MSCs may involve the inhibition of the EMT process. This supports the notion that BM-MSCs exert an anti-cancer effects *in vivo* by inhibition EMT and suppression of a migratory/invasive phenotype, all of which are considered to be early steps in the metastatic process [39–42, 48–50]. Our results demonstrating that hBM-MSC-CM inhibits TGF-β-induced EMT in SCC cells, suggested that BM-MSCs contain factors that antagonize TGF-β's pro-metastatic effects. This finding coupled with our other result indicating that CD109, a known TGF-β antagonist, is expressed on BM-MSCs and released into the culture medium provided the impetus to examine whether CD109 mediates the anti-TGF-β and anti-malignant activity of hBM-MSC-CM.

Our results demonstrating that knockdown of CD109 in hBM-MSCs abrogates the inhibitory effects of hBM-MSC-CM on EMT, migration, invasion and spheroid formation in SCC cells, indicate that CD109 released from hBM-MSCs is essential for these anti-tumor effects. Altogether, our results suggest that hBM-MSC-CM possesses potent anti-cancer activity and that it involves CD109-mediated inhibition of EMT and stemness. The anti-cancer effect of CD109 is further supported by our results showing that overexpression of CD109 in A431 cancer cells effectively attenuates the basal and TGF-β-induced EMT responses and suppresses migration and invasion of these cells.These findings demonstrate that CD109 mediates the anti-cancer effects of hBM-MSC-CM such as inhibition of EMT, migration and invasion, are consistent with our previous report that CD109 is a strong inhibitor of TGF-β signaling [19–21, 23].

Further evidence for the anti-cancer effect of hBM-MSC on SCC cells is provided by our results showing that hBM-MSC-CM markedly decreases cancer cell stemness markers and spheroid formation in A431 and FaDu cells *in vitro*, indicating a decrease of cancer stem cell-like cells in their population. The critical role of CD109 in mediating these effects of hBM-MSC-CM is supported by the observation that siRNA-mediated silencing of CD109 in hBM-MSCs significantly abrogates those effects. Furthermore, our finding that overexpression of CD109 in A431 cells leads to a significant decrease in SOX2 expression and a concomitant decrease of spheroid formation in these cells, supports the notion that CD109 plays a fundamental anti-cancer role in SCC cells, and that it mediates the anti-cancer effect of hBM-MSC-CM in SCC cells.

**Figure 7:**
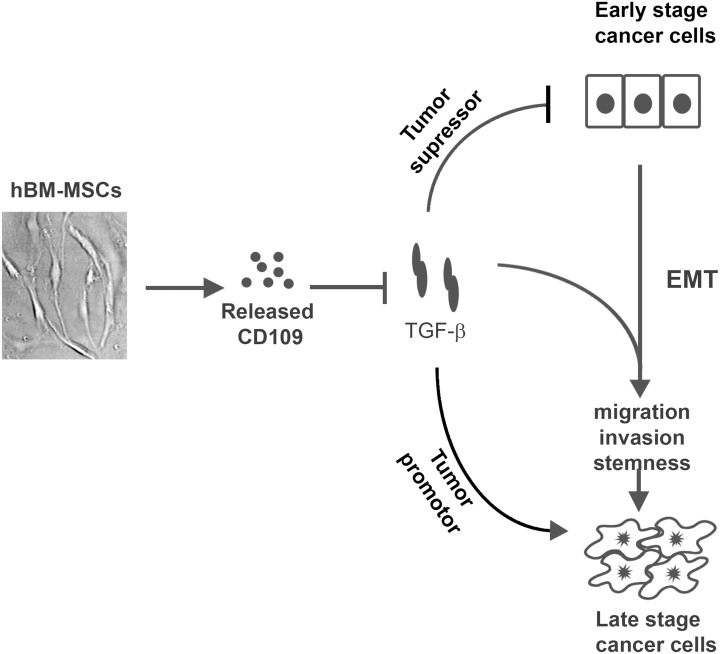
A schematic model depicting the possible mechanism underlying the anti-cancer effect of hBM-MSCs-CM TGF-β has a dual function in tumor progression, by acting as a tumor suppressor in early stage of cancer, and then switching its function to that of a tumor promoter in late stages of cancer. hBM-MSCs release CD109 into the conditioned medium. In the context where TGF-β acts as a tumor suppressor, CD109 released from BM-MSC would inhibit TGF-β's tumor suppressor function favoring tumor progression. However, in the context of the current study which mimics advanced state of cancer where TGF-β acts as a tumor promoter, CD109 in the hBM-MSC-CM would inhibit EMT, migration, invasion and stemness, suppressing TGF-β‘s pro-metastatic effects. Thus, the discrepant results with BM-MSCs from various laboratories and under different experimental settings, showing both pro- and anti-cancer effects are possibly related to the paradox of TGF-β action in cancer and CD109 as a TGF-β inhibitor.

There is discrepancy in the literature regarding the role of hBM-MSCs in cancer with some studies reporting tumor promoting effects [35, 36], while other suggesting anti-cancer effects [37, 38, 51, 52]. In addition, there is limited information regarding the molecular interactions between hBM-MSCs and cancer cells [53, 54]. Among the large array of cytokines and other factors produced by hBM-MSCs that can influence cancer cell behavior, TGF-β is noteworthy in that it has a broad spectrum of potent effects and plays a paradoxical role in cancer progression. TGF-β acts as a tumor suppressor in benign tissue and early stage cancers, but in late stages of cancers it acts as a tumor promoter and pro-metastatic molecule [12–16, 55]. These discrepant results reported on the role of BM-MSCs on cancer cell behaviour can be partially explained by the role of CD109 as a strong TGF-β antagonist [19–23], inhibiting the dual effects of TGF-β on cancer cells. Our findings from the current study demonstrate that CD109 is expressed by hBM-MSCs and that it is released into culture media. Although the expression of CD109 has previously been reported on BMSCs, its function in these cells has remained unknown. The current study demonstrates that the released CD109 from hBM-MSCs can inhibit TGF-β signalling, EMT response, migration, invasiveness, and stemness in SSC cells. Our findings suggest that CD109 inhibits TGF-β's tumor promoter function and supresses the malignant traits of cancer cells under the setting of our experimental conditions. However, we cannot rule out the possibility that CD109 may act as a tumor promoter under conditions - differing from those of the current experimental setting. Therefore, in the context in which TGF-β acts as a tumor suppressor, CD109 would inhibit the tumor suppressor function of TGF-β, endowing hBM-MSCs with ability to favor tumor growth and progression. In the context of the current study, we use *in vitro* SSC cells in culture, which is thought to mimic advanced stages of cancer. Our results reveal that CD109 released from hBM-MSCs supresses TGF-β induced EMT, invasion and stemness in SSC cells. This is consistent with the notion that TGF-β functions as a tumor promoter in late stage cancer and indicates that CD109 may partially mediate the anti-metastatic activity of hBM-MSC-CM. Figure 7 shows a schematic model of the possible mechanism for the anti-cancer effect of hBM-MSC-CM. In this model, we propose for first time that CD109 released from hBM-MSCs could, at least partially, account for the anti-cancer effect of hBM-MSC-CM, and that CD109 action is linked to inhibition of TGF-β-induced pro-metastatic responses, leading to suppression of EMT, attenuation of migration and invasion, and decrease in stemness, in cancer cells.

## MATERIALS AND METHODS

### Cell culture

The human bone marrow mesenchymal stem cells (hBM-MSC) were purchased from Lonza. The human squamous carcinoma cell A431 (Cat. No. ATCC^®^ CRL-1555™) and FaDu (ATCC^®^ HTB-43™) cell lines were purchased from the American Type Culture Collection. The cell lines were cultured in Dulbecco's modified Eagle's medium (DMEM; Gibco Life Technologies, USA Cat No. 11995-065) supplemented with 10% fetal bovine serum (FBS; Gibco Life Technologies, Canada; Cat. No.12483-020) at 37°C in 5% CO_2_. A431 cells stably expressing CD109 (or its empty vector, EV) were generated as described in [21].

### Preparation of the hBM-MSC-conditioned medium and co-culture with tumor cells

The hBM-MSCs and human fibroblast cells were cultured in DMEM/10 % FBS to 90% confluence, and were switched to serum free DMEM for an additional 48 h. Serum free conditioned medium from hBM-MSCs was centrifuged at 1200 rpm g at 4°C for 10 min and then at 2000 rpm at 4°C for another 20 min. The supernatants were filtered by 0.22-μm nylon filters and stored at -80°C until use. This was designated as hBM-MSC-CM and human fibroblast-CM, respectively. For the pre-treatment of tumor cells with hBM-MSC-CM, the cancer cells were washed three times with phosphate-buffered saline, and incubated with hBM-MSC-CM at 37°C in 5% CO2 for three days prior to be use in the subsequent experiments.

### Western blot analysis

The proteins were extracted from the whole cell lysates using RIPA cell lysis buffer and the protein concentration was determined. In total, 20 μg of the extracted total cellular protein from each sample were separated via SDS-PAGE, and transblotted onto EMD Millipore Immobilon™-P PVDF Transfer Membranes (EMD Millipore Cat No.: IPVH00010, USA). Western blot analyses were conducted with the following antibodies: mouse monoclonal anti-CD109 (Cat. No. 556039, BD Biosciences), anti-fibronectin (Cat. No.610078, BD Biosciences), anti-β-actin antibodies (Cat. No. sc-47778, Santa Cruz Biotechnology), rabbit polyclonal anti-Snail (Cat. NO. ab5351, abcam), anti-twist (Cat. No. ab505181; abcam), anti-Sox2(Cat. No: ab137385, abcam), and anti-Slug (Cat. No. 9585 S Cell signaling).

### siRNA transfections

hBM-MSCs were transfected with CD109 siRNA (ID# s43924), or a negative control siRNA(ID#4611) (Ambion, life technologies) using Lipofectamine 2000 (Cat. No. 1136666 Invitrogen) according to the manufacturer's instructions.

CD109 siRNA sequence:

Sense: 5’->3’: GAUCUAUCCAAAAUCAAGAtt.

Antisence: UCUUGAUUUUGGAUAGAUCtt

Negative Control siRNAs are designed to have no known target in the cells being used. They are important for distinguishing sequence-specific silencing from non-specific effects in the RNAi experiment.

### Flow cytometric assay for apoptosis using annexin V and propidium iodide

A431 cancer cells were plated on 6-well plates at 8000 cells per well in the serum-free hBM-MSC-conditioned medium or serum free human fibroblast cells conditioned medium or DMEM as control. This experiment was carried out consecutively for three days and was performed three times (n= 3) with triplicates each time. 72 hours after incubation, cancer cells were trypsinized for 10 minutes at 37°C. Dissociated cells were washed with phosphate-buffered saline (PBS) two times, re-suspended in 500μL binding buffer, and then incubated for 1 hour at room temperature in the presence of 0.5 μg/mL FITC-Annexin V apoptosis detection Kit (Cat. No. 556547, BD bioscience) and 5μL propidium iodide (Cat. No. 25535-16-4, Sigma) for 5 minutes in binding buffer as described by the manufacturer. After incubation, the cells were analyzed by flow cytometry.

### Wound healing assay

A431 cancer cells were seeded at a density of 6×10^5^ cells/well into Costar^®^ 6 Well Clear TC-Treated Multiple Well Plates (Product #3516, Corning Inc, USA) and cultured for ~48 h or until the cells had reached ~90% confluency. Cells were then were pre-incubated with serum-free medium (SFM) for 24h to inhibit cell proliferation [56]. The monolayer of A431 cells were scratched across the centre with a sterile 200 μl pipette tip to create a cell-free line. The culture medium was aspirated and washed three times to remove cellular debris. The culture plates were replenished with serum free DMEM, human fibroblast-CM and hBM-MSC-CM in the absence or presence of 100pm TGF-β1 (Cat. No. 7754-BH-005, R&D). Samples were taken at the beginning and at 24 h after culture, with 5% CO_2_ at 37°C. Photographs were taken immediately (0 h) and 24 h after the scratch and Image-J software was used to measure the width of the wound area. The experiments were repeated 3 times. Cell migration was expressed as percentage of the scratch area filled by migrating cells at 24 h post scratch: migration rate=(T0 hr scratch width - T24 hr scratch width)/T0 hr scratch width) ×100%.

### Transwell migration assay and matrigel invasion assay

For the transwell migration assay, 5×10^4^ cancer cells/well were plated in the upper wells and each well was filled with 500 μl hBM-MSC-CM, human fibroblast-CM and DMEM. In the lower chamber, DMEM supplemented with 10% FBS that served as a chemoattractant to drive cellular migration. The cells were incubated for 24 h at 37°C, and 10% FBS served as the control. The cells that did not migrate were removed using a cotton swab. The cells that did migrate were stained with crystal violet (Cat. No. CAS 548-62-9, Fisher scientific) and then counted under a microscope (EVOSXL CORE, Life technologies). In total, three views were chosen at random, and each experiment was repeated independently in triplicate.

The Matrigel invasion assay was done by using the BD Biocoat Matrigel Invasion Chamber (Cat. NO. 354480; pore size: 8 mm, 24-well; BD Biosciences) according to the manufacturer's protocol. Briefly, the control inserts or matrigel-coated inserts were rehydrated with plain DMEM for 2 h before use. Cells (5×10^4^ cells) in 500ul Dulbecco's modified Eagle's medium without serum were seeded on the upper chamber; the lower chamber was filled with DMEM supplemented with 10% FBS as chemoattractant. After 24 hours, cells on the upper side of the membrane were wiped off; cells on the lower side of the membrane were fixed for 10 min by cold methanol and stained with 0.1% crystal violet solution for 20 min and washed with PBS 3 times. Cell numbers were counted using an inverted microscope at ×200 magnification with 10 fields of view, and the mean values were taken as the invasive cell number. All assays were done in triplicate for at least three independent experiments.

### Tumor sphere formation assays

A431 cancer cells were treated with hBM-MSC-CM or human fibroblast-CM as well as control media (DMEM) for 4 days, then were trypsinized, washed and passed through a 40 μM cell strainer to obtain single cell suspension. Cells were re-suspended into a six-well ultra-low attachment plate (Cat NO. 29443-030VWR) at 10,000 single cells/well and cultured in DMEM/F12 medium with 20 ng/ml hEGF, 20 ng/ml hbFGF, and 2% B-27 (Life Technologies Corp.) at 37°C in 5% CO_2_ (serum free spheroid medium). Medium was changed once a week. Two weeks later, individual spheres were counted under an inverted microscope at 40x magnification. The percentage of cells capable of forming spheres was calculated as follows: [(number of spheres formed/number of cells plated) × 100].

### Statistical analysis

All values are expressed as mean of at least 3 independent experiments ± SD. Comparisons between two groups were analyzed by a two-tailed Student’s-test, and comparisons between more than two groups were analyzed by one-way ANOVA. A value of P < 0.05 was considered statistically significant. All analyses were performed with Prism Graph pad.

## CONCLUSION

Our findings indicate that hBM-MSCs inhibit the malignant conversion of SSC cells by a paracrine effect via released factors and link the cancer inhibitory effect of hBM-MSCs to the TGF-β co-receptor, CD109. This represents the first report linking the cancer inhibitory effect of hBM-MSCs to CD109, and CD109-mediated suppression of TGF-β-induced EMT, migration, invasion and stemness in cancer cells. Our results provide a mechanistic basis for the inhibitory effect of hBM-MSC-CM on metastatic properties of cancer cells and provide a new insight into the potential of the hBM-MSC-CM to prevent cancer metastasis.

## SUPPLEMENTARY MATERIALS FIGURES



## Abbreviations:

hBM-MSC-CM: human bone marrow mesenchymal stem cells conditioned medium
hFibro-CM: human fibroblast cells conditioned medium
EMT: epithelial to mesenchymal transition
SCC: squamous cell carcinoma
