# Supplementary material for: CD109 released from human bone marrow mesenchymal stem cells attenuates TGF-β-induced epithelial to mesenchymal transition and stemness of squamous cell carcinoma
Source: Oncotarget. 2017 Sep 16;8(56):95632–47. doi: 10.18632/oncotarget.21067 (PMC5707049; doi:10.18632/oncotarget.21067)
Supplement: Supplementary file 1 [file oncotarget-08-95632-s001.pdf]

CD109 released from human bone marrow mesenchymal stem cells attenuates TGF-β-induced epithelial to mesenchymal transition and stemness of squamous cell carcinoma

SUPPLEMENTARY MATERIALS

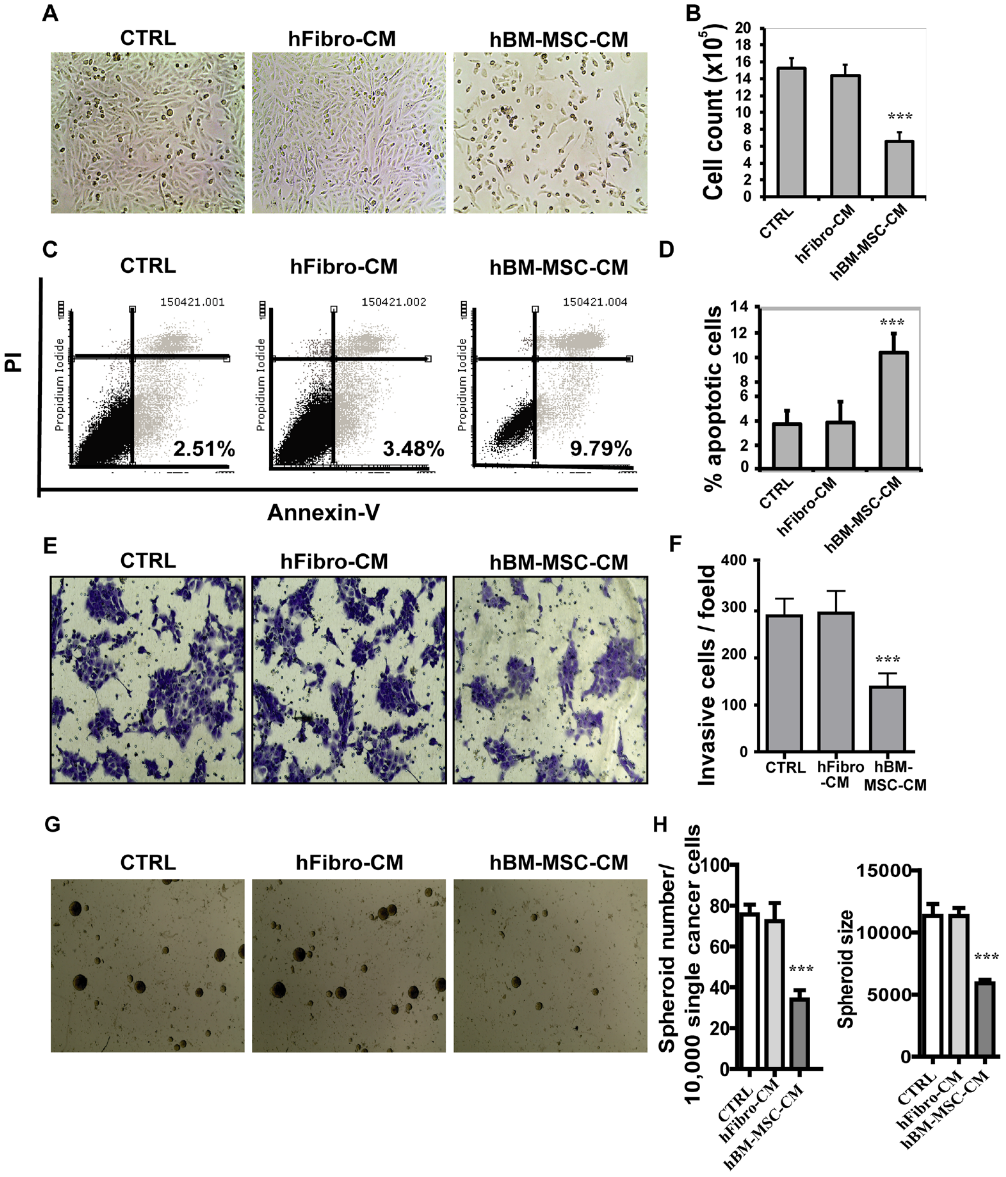

Supplementary Figure 1: The effect of hBM-MSCs-CM on FaDu, a model cell line of head and neck squamous cell carcinoma. **(A)** Light microscope images of FaDu cells culture in different media as indicated. FaDu cancer cells were treated with hBM-MSC-CM, human fibroblast-CM and DMEM (CTRL) for 72 hrs **(B)** Qualification of cell growth in experiment (A). hBM-MSC-CM markedly inhibited the proliferation of FaDu cells. **(C)** Flow cytometry analysis of apoptosis by Annexin V and PI staining. FaDu cells were treated as described before, then analyzed by FACSCalibur flow cytometer. **(D)** Qualification of apoptosis assay in experiment (C). hBM-MSC-CM treatment significantly increased cancer cells apoptosis. **(E)** Representative image for Matrigel invasive assay. **(F)** Quantification of the Matrigel invasive assay. Invaded cells were stained by 1% crystal violet, photographed and counted. hBM-MSC-CM significantly supressed cancer cells invasion. **(G)** Representative image for tumor spheroid assay. **(H)** Quantification of the tumor spheroid assay. Five random fields (x100) for each condition were photographed, and the sphere numbers were counted. hBM-MSC-CM significantly supressed cancer cells capacity to form spheroids. All the results are shown as mean ± SD from at least three independent experiments. Significance is calculated using a one-way ANOVA; **P<0.01 and ***P<0.001. Magnification, ×100.

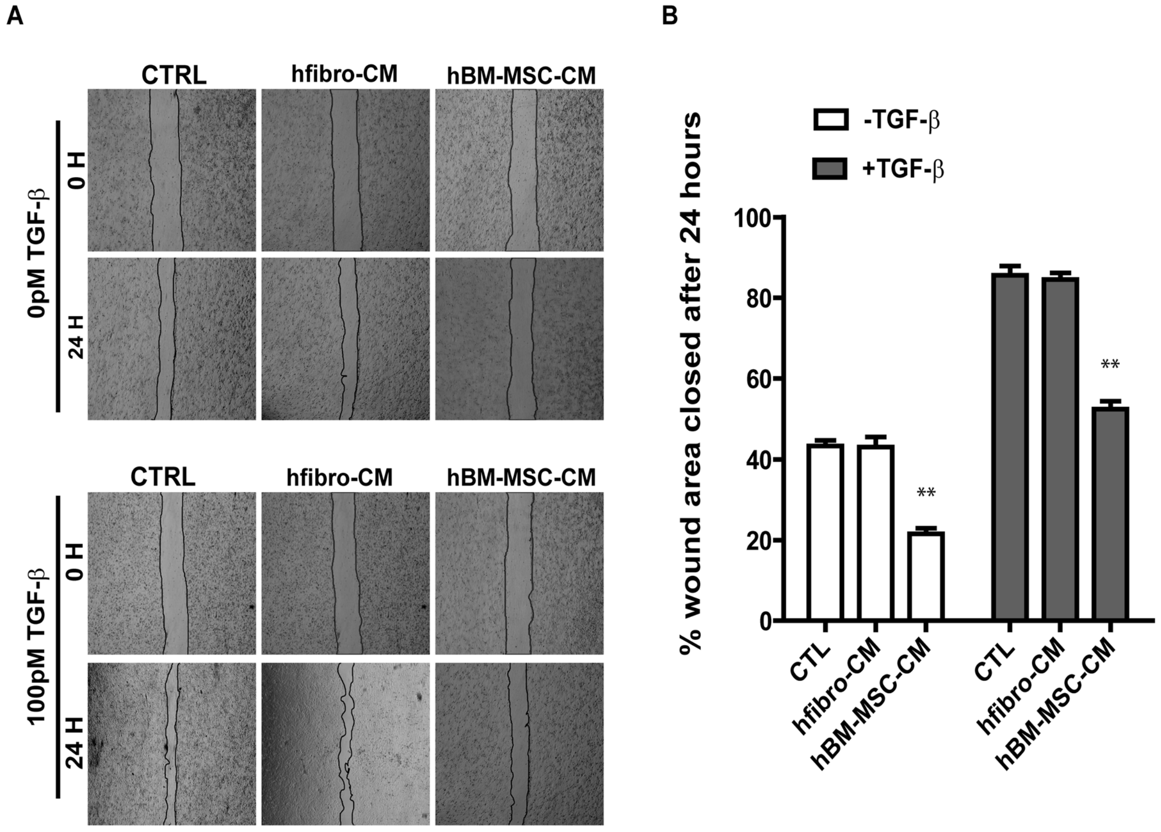

Supplementary Figure 2: hBM-MSC-CM significantly inhibits the migration of FaDu cells. Representative images **(A)** and quantification **(B)** of wound-healing assays on FaDu cells treated with hBM-MSC-CM, human fibroblast-CM and DMEM (CTRL) for 72 hrs. Cell migration is expressed as percentage of the scratch area filled by migrating cells at 24 h post scratch: migration rate = (T0 hr scratch width - T24 hr scratch width)/ T0 hr scratch width) ×100%. hBM-MSC-CM significantly supressed cancer cells wound healing ability. All the results are expressed as the mean ± S.D. of three independent experiments. Significance is calculated using a one-way ANOVA; * $P < 0.05$. ** $P < 0.01$ and *** $P < 0.001$. Magnification, ×100.
